# Myocardial, Valvular, and Vascular Abnormalities in Lipedema

**DOI:** 10.31083/RCM50869

**Published:** 2026-09-03

**Authors:** Attila Nemes

**Affiliations:** ^1^Department of Medicine, Albert Szent-Györgyi Medical School, University of Szeged, 6720 Szeged, Hungary

**Keywords:** myocardial, vascular, valvular, lipedema

## Abstract

Lipedema is a chronic, progressive, and often underdiagnosed disease characterized by the pathological, bilateral, and symmetrical accumulation of adipose tissue. This distribution results in disproportionate and painful swelling of the limbs. Typically extending distally from the hips, this predominantly female condition is frequently misidentified as lifestyle-induced obesity or bilateral lymphedema. This review aims to provide a comprehensive overview of the current understanding of structural and functional abnormalities in myocardial dimensions, mechanics, valves, and major arteries in patients with lipedema. According to the existing literature, left atrial enlargement, left ventricular rotational abnormalities, mitral annular dilation, and increased aortic stiffness have been identified in patients with lipedema. These findings suggest latent but significant alterations in left heart physiology. Although current data remain limited and are primarily focused on the left heart and the aorta, the rapid evolution of cardiac imaging technologies suggests that both clinical knowledge and the body of research in this field are poised for significant expansion.

## 1. Introduction

Lipedema is a chronic and progressive, yet frequently underdiagnosed, clinical entity characterized by the pathological accumulation of adipose tissue. This distribution results in disproportionate and painful enlargement of the lower and/or upper extremities. The clinical presentation typically involves bilateral, symmetrical adipose tissue accumulation extending distally from the hip region. The condition exhibits a marked female predominance, and is frequently misidentified as obesity or primary or secondary lymphedema. Pathognomonic features include the ‘cuff sign’ (distinct sparing of the pedal region), non-pitting edema, increased capillary fragility leading to easy bruising, and localized pain. The extant literature suggests a multifactorial etiology, involving polygenic susceptibility alongside hormonal, microvascular, and lymphatic dysregulation [1,2,3,4,5,6,7,8].

The exact incidence of lipedema remains unknown**, **primarily due to widespread underdiagnosis, misdiagnosis as lifestyle-induced obesity or lymphedema, and the lack of definitive diagnostic biomarkers. Epidemiological studies suggest a high, yet widely varying, prevalence of lipedema, with estimates ranging from a conservative 0.001% in clinical settings to over 10% in broad literature reviews [9,10]. While a 2020 review suggests 10–11% of women (approx. 400 million) are affected [9], published estimates of the prevalence of lipedema range from 5% to 15% [11,12,13,14]. The wide discrepancy between studies, such as the low 0.001%, likely stems from systemic underdiagnosis and methodological differences in assessment [10]. The condition occurs almost exclusively in women, typically triggering or exacerbating during periods of significant hormonal fluctuation, such as puberty, pregnancy, or menopause. In men, lipedema is exceedingly rare and is strictly associated with severe hormonal imbalances, or other pathological states [1,2,3,4,5,6,7,8].

Regarding therapy, the adipose deposits in lipedema are frequently refractory to conventional caloric restriction and dietary interventions. Medical compression stockings (MCSs) are established and widely accepted components of the multimodal management of lipedema. Patients diagnosed with lipedema frequently undergo complex decongestive therapy (CDT), using custom-made, flat-knit compression garments (Compression Class 2) to maintain limb volume reduction during the maintenance phase. Pantyhose-style garments represent an optimal compression modality, as they provide comprehensive coverage of all affected anatomical regions, while the structural properties of flat-knit materials ensure superior adaptation to the irregular contours of the lower extremities. Notwithstanding these conservative approaches, liposuction remains the most efficacious therapeutic intervention currently available [15,16].

Although patients with lipedema generally display a less severe cardiovascular profile, the available literature regarding lipedema-associated abnormalities in cardiac chamber volumes and function, valvular structure, and vascular morphology remains extremely limited. This review aims to synthesize the existing literature on myocardial, valvular, and vascular morphology and functional impairments associated with lipedema. Although current evidence remains sparse, advancements in cardiovascular imaging indicate that research in this field is poised for significant expansion. With respect to vascular abnormalities, only major vessels, such as the aorta and pulmonary arteries, are presented when data is available.

## 2. Cardiovascular Imaging and MAGYAR-Path Study

In recent years, the integration of cardiac magnetic resonance imaging and cardiac computed tomography into standard clinical workflows has been driven by advances in imaging hardware and computational software. Furthermore, echocardiography has undergone significant evolution, with the emergence of novel techniques that are now commonplace in clinical practice [17]. These advances enable more precise measurements of chamber volumes and function, detailed assessment of myocardial mechanics, a deeper understanding of valvular structure and performance, and more comprehensive evaluation of vascular health. Speckle-tracking echocardiography (STE) and three-dimensional (3D) echocardiography, as well as their integration into three-dimensional speckle-tracking echocardiography (3DSTE) represents cutting-edge diagnostic developments that facilitate a comprehensive evaluation of both chamber dimensions and valvular functional characteristics through the analysis of virtually acquired 3D datasets [18,19,20,21,22,23,24,25].

A structured literature search has been conducted, but the clinical data regarding this topic are severely limited. Most of the myocardial, valvular, and vascular findings associated with lipedema presented in this summary are derived from the ‘Motion Analysis of the heart and Great vessels bY three-dimensionAl speckle-tRacking echocardiography in Pathological cases’ (MAGYAR-Path) Study [26,27,28,29,30,31]. This clinical study was organized at the University of Szeged (Hungary) as a result of a cooperative effort between the Department of Dermatology and Allergology and the Department of Medicine. The study cohort comprised patients with lipedema receiving clinical care at the Department of Dermatology and Allergology. Participants underwent a detailed cardiological evaluation, which included medical history, physical examination, electrocardiography, and both two-dimensional (2D) Doppler echocardiography and 3DSTE imaging. For comparative analyses, an age- and sex-matched control group of healthy subjects was established, excluding individuals with conditions that could potentially confound the cardiovascular findings. In all cases, echocardiographic examinations were performed using a Toshiba Artida® system (Toshiba Medical Systems, Tokyo, Japan) equipped with a PST-30BT transducer for 2D Doppler imaging, and attached to a PST-25SX matrix-array transducer for 3DSTE-derived data acquisition. Data processing was performed using a 3D Wall Motion Tracking software (version 2.7, UltraExtend, Toshiba Medical Systems). The MAGYAR-Path Study was launched in 2011 to delineate the diagnostic and prognostic utility of 3DSTE-derived parameters across various conditions, with a specific focus on lipedema [22,23,24,25,26,27,28,29,30]. 

## 3. The Left Heart and the Aorta

### 3.1 Left Ventricle (LV)

#### 3.1.1 Normal Physiological Conditions

The LV is characterized by an ovoid or bullet-like shape. Accurate contouring for quantitative assessment may be challenging due to the presence of papillary muscles and muscle bands. In the physiological state, ventricular systole is characterized by mitral valve (MV) closure and aortic valve (AV) opening, facilitating blood ejection from the LV into the aorta. Conversely, during diastole, AV closure and MV opening allow for LV filling from the left atrium (LA). The LV wall consists of three fiber layers: subepicardial fibers (left-handed orientation), mid-layer fibers (circumferential orientation), and subendocardial fibers (right-handed orientation) [32,33,34,35,36]. LV motion is a complex 3D process involving radial thickening, longitudinal shortening, and circumferential narrowing in systole. These deformations are quantified using echocardiographic strain analysis, including radial strain (LV-RS), longitudinal strain (LV-LS), and circumferential strain (LV-CS), which may be combined into area strain (LV-AS) or comprehensive 3D strain (LV-3DS). Additionally, the LV exhibits a wringing-like motion; during systole, the LV base rotates clockwise, while the LV apex rotates in the opposite counterclockwise direction, creating LV twist [32,33,34,35,36]. The absence of normal LV rotational mechanics, characterized by both the LV apex and base rotating in the same direction (either clockwise or counterclockwise), is defined as LV ‘rigid body rotation’ (RBR) [37]. The diagnosis of reversed LV rotational mechanics is established when LV base rotates counterclockwise, while LV apex rotates clockwise. During diastole, the heart undergoes untwisting as these regions return to their original positions [32,33,34,35,36] (Fig. 1).

**Fig. 1.**
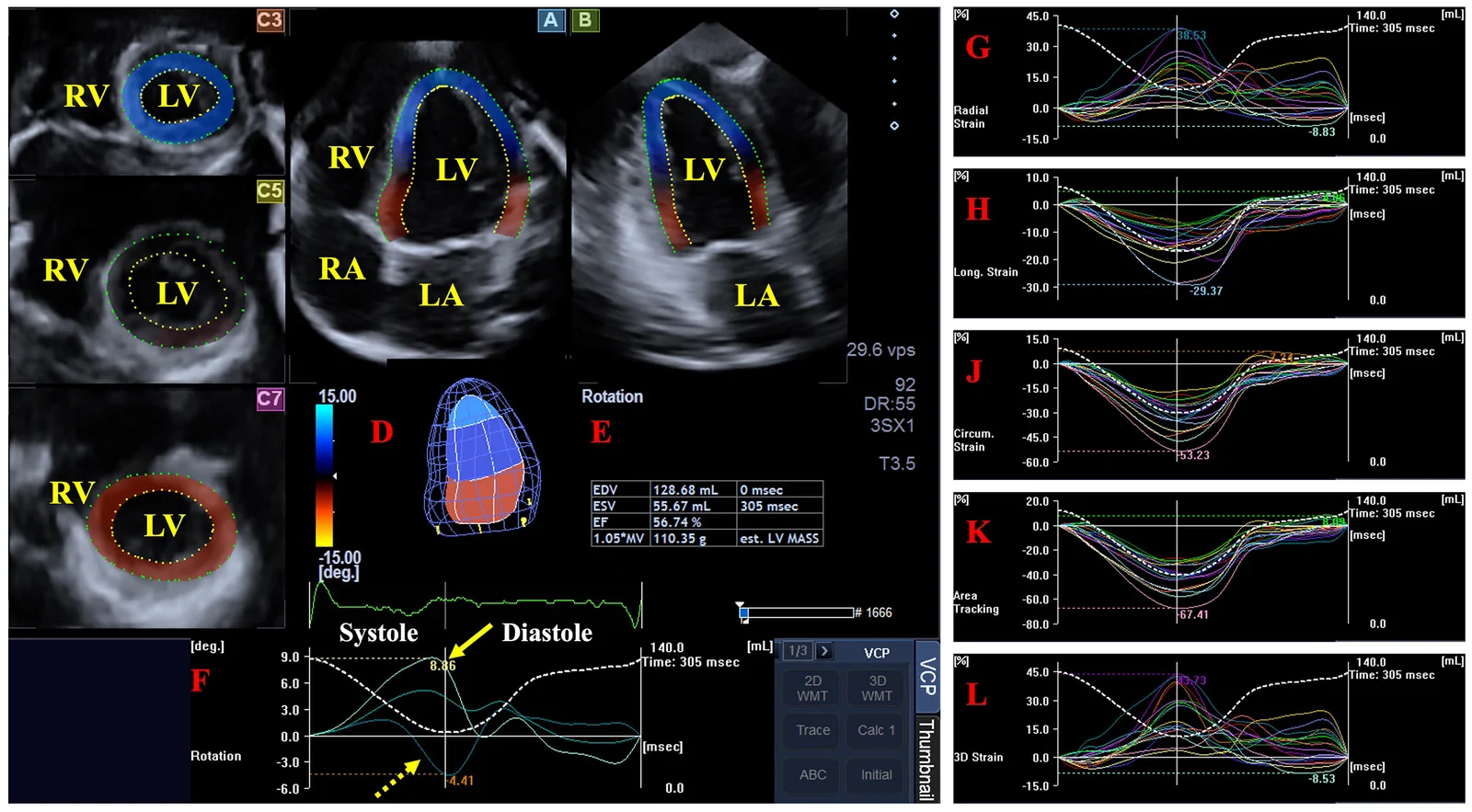
**Three-dimensional (3D) speckle-tracking echocardiographic assessment of left ventricular (LV) parameters in a representative case**. Following 3D echocardiographic dataset acquisition, apical four-chamber (A) and two-chamber (B) long-axis views, along with short-axis views at the apical (C3), midventricular (C5), and basal (C7) LV levels, are reconstructed using a dedicated software. In addition to a 3D virtual model of the LV (D), calculated LV volumetric data and LV ejection fraction (E) are displayed. The figure also shows apical (yellow arrow) and basal (dashed yellow arrow) LV rotations (F), alongside time-resolved curves for LV global (white curve) and segmental (colored curves) radial (G), longitudinal (H), circumferential (J), area (K), and 3D (L) strains. A curve representing temporal LV volume changes (dashed white curve) is also provided. EDV, end-diastolic volume; ESV, end-systolic volume; EF, ejection fraction; LA, left atrium; RA, right atrium; LV, left ventricle; RV, right ventricle.

#### 3.1.2 In Lipedema

No studies have reported deterioration of routine M-mode or 2D echocardiographic parameters in patients with lipedema. Using 3DSTE, which allows more advanced assessment of LV function within the framework of the MAGYAR-Path Study, lipedema was found to be associated with significant abnormalities in LV rotational mechanics. In some lipedema cases, near-complete absence of LV twist (one out of 25 cases), counterclockwise LV-RBR (one out of 25 cases), and reversed LV rotational mechanics (one out of 25 cases), could be detected [26]. The exact prevalence of these abnormalities among healthy individuals or in specific pathologies is unknown; however, literature indicates that 6% of healthy subjects exhibit LV-RBR. LV-RBR is known to occur most frequently in non-compaction cardiomyopathy (approximately 50–100%) and cardiac amyloidosis (approximately 60%) [37]. In lipedema patients with normally directed LV rotational mechanics, reduction of apical LV rotation and consequent reduction in LV twist could be observed in the presence of preserved basal LV rotation. When direct comparisons were performed with patients with lymphedema, similar abnormalities were not detected in patients with normally directed LV rotational mechanics, suggesting that these findings may represent a novel differential diagnostic feature distinguishing lipedema from lymphedema [26]. Following 60 minutes of compression garment use, basal LV rotation was significantly reduced, while apical LV rotation significantly increased, resulting in unchanged LV twist in lipedema patients with normally directed LV rotational mechanics. This pattern may, in part, reflect an adaptive mechanism underlying lipedema-related LV volume changes. In lipedema patients with marked LV rotational abnormalities, different patterns of LV rotational changes could be detected [27]. In another study, lipedema was associated with significantly higher global and mean segmental LV-CS and LV-AS, with unchanged LV-RS, LV-LS and LV-3DS compared with healthy individuals. These elevated strain values are considered to help preserve LV ejection fraction despite underlying cardiac abnormalities such as rotational impairments. Short-term use of MCSs showed no significant global effect on LV strain parameters. While global deformation remained largely unchanged, some regional parameters demonstrated improvement [28].

### 3.2 Left Atrium (LA)

#### 3.2.1 Normal Physiological Conditions

The LA is composed of various muscle bands, such as the interatrial and septoatrial bands, which are anchored to the rim of the oval fossa. The LA acts as a dynamic regulator of LV filling through three roles: a reservoir (collecting pulmonary venous return during LV systole), a conduit (passive passage of blood during early LV diastole), and a booster pump (active contraction completing LV filling during late diastole). LA volume peaks at the end of systole (just before the MV opens) and reaches its minimum at the end of diastole (when the MV closes). Functional efficiency is assessed by calculating various stroke volumes and emptying fractions derived from these volume changes. Like the ventricle, the LA follows the Frank-Starling mechanism, where increased stretching (preload) enhances contractility up to a physiological limit. The LA walls exhibit phasic contraction and relaxation dynamics analogous to those of the LV, however LA deformation patterns are characterized by strain directions opposite to those observed in the LV [38,39,40,41] (Fig. 2).

**Fig. 2.**
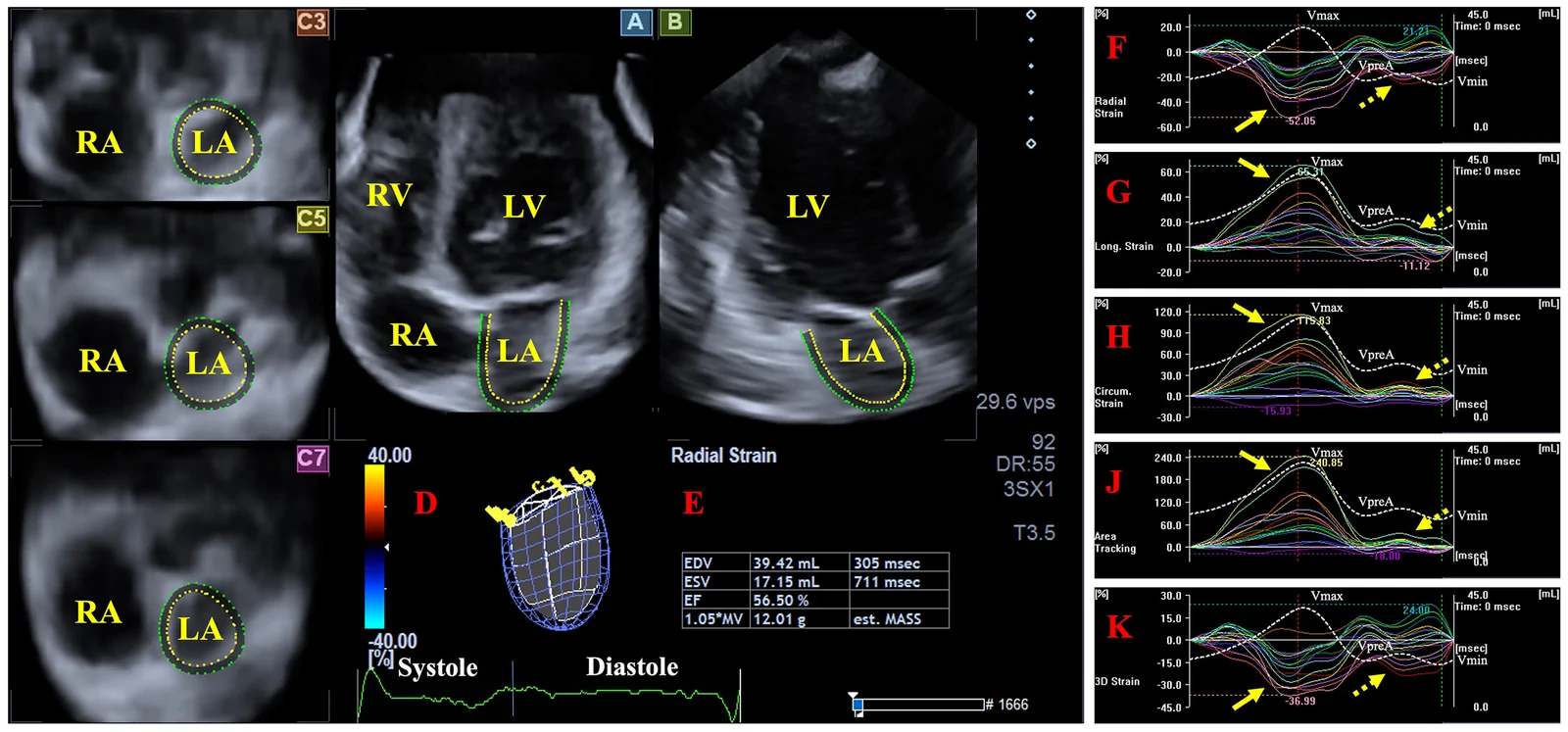
**Three-dimensional (3D) speckle-tracking echocardiographic assessment of left atrial (LA) parameters in a representative case**. Following automatic reconstruction of apical four-chamber (A) and two-chamber (B) long-axis views with short-axis views at the basal (C3), midatrial (C5), and superior (C7) LA levels, a virtual 3D cast of the LA (D) together with LA volumes (E) are displayed. The figure also shows time-global (white curve) and segmental (colored curves) radial (F), longitudinal (G), circumferential (H), area (J), and 3D (K) LA strain curves, as well. A curve representing temporal LA volume changes (dashed white curve) is also provided with end-systolic maximum (Vmax), early diastolic pre-atrial contraction (VpreA) and late diastolic minimum (Vmin) LA volumes. Yellow arrow represents end-systolic peak LA strains, while yellow dashed arrow represents LA strains at atrial contraction. EDV, end-diastolic volume; ESV, end-systolic volume; EF, ejection fraction; LA, left atrium; RA, right atrium; LV, left ventricle; RV, right ventricle.

#### 3.2.2 In Lipedema

Patients with lipedema exhibited significantly larger LA volumes compared to healthy controls across all phases of the cardiac cycle, as demonstrated by findings from the MAGYAR-Path Study. Following the one-hour application of MCSs, these LA volumes demonstrated a further increase. Correspondingly, the total, passive, and active LA stroke volumes (TASV, PASV, and AASV, respectively)—representing all phases of LA function—were elevated in lipedema patients, with additional increases observed during systole and early diastole post-MCS application. While systolic total atrial emptying fraction (TAEF) remained unchanged both at baseline and after MCS use, the passive atrial emptying fraction (PAEF) increased following MCS application compared to resting values. The active atrial emptying fraction (AAEF) was elevated in patients with lipedema at rest, though it remained unaffected by short-term MCS use. Regarding LA deformation mechanics, peak global LA-RS and LA-3DS were higher in lipedema patients than in controls; however, these parameters remained stable, showing no significant alterations following one hour of MCS use. Conversely, peak global LA-LS significantly increased post-MCS application compared to resting measurements. While no significant differences were observed in global LA strains during atrial contraction between the study cohorts, segmental strains exhibited specific alterations. Finally, while LA-CS was increased in patients with lipedema and remained stable after MCS use, LA-3DS showed a increase following one hour of compression therapy [29].

### 3.3 Mitral Valve (MV)

#### 3.3.1 Normal Physiological Conditions

The MV is a complex, saddle-shaped 3D structure consisting of the mitral annulus (MA), two leaflets (anterior and posterior), chordae tendineae, and papillary muscles. Its primary role is to ensure unidirectional blood flow from the LA to the LV during diastole, while preventing retrograde flow into the atrium during systole. Proper valve closure depends on the coordinated contraction of the surrounding atrial and ventricular tissue [42,43,44,45] (Fig. 3).

**Fig. 3.**
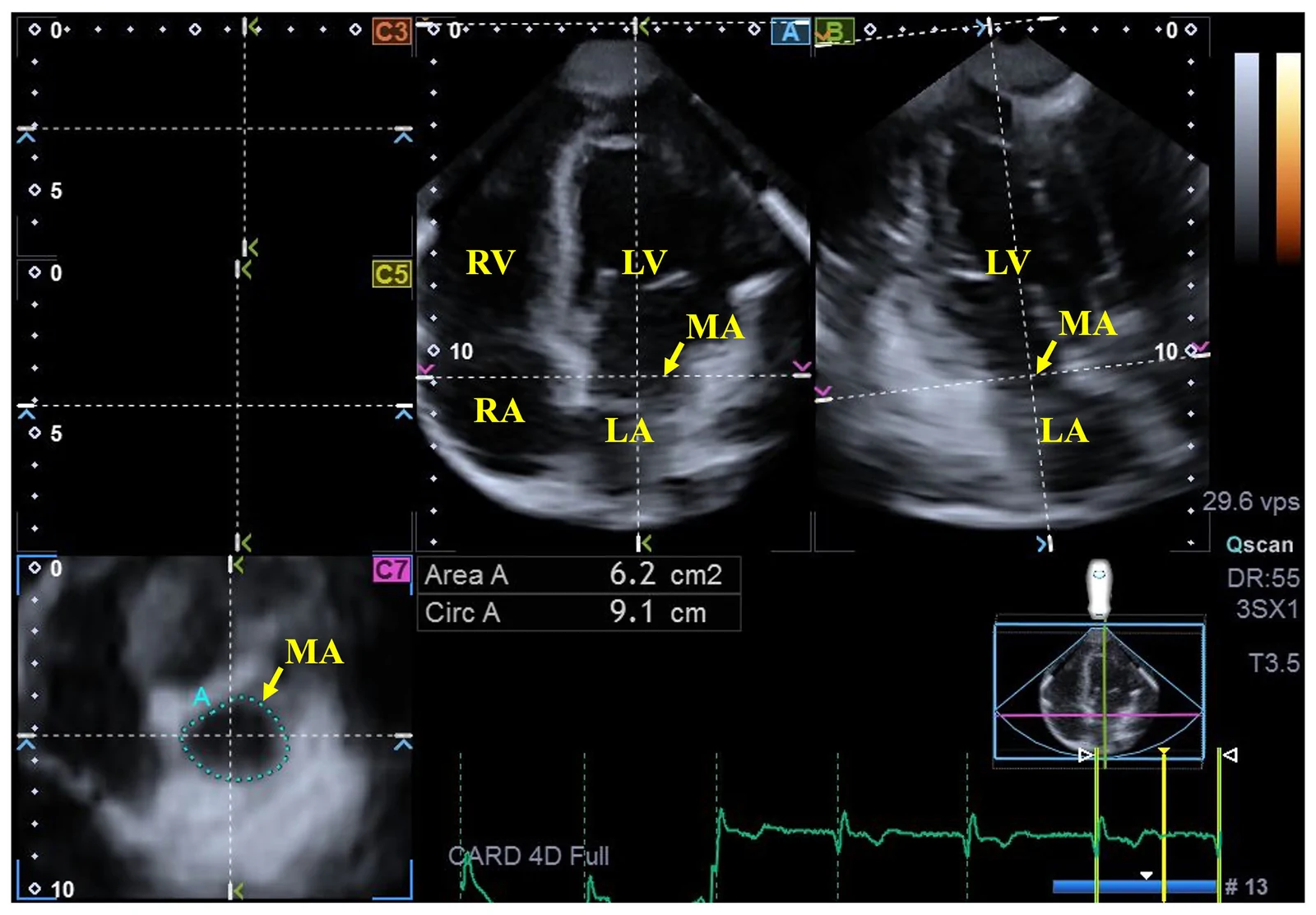
**Assessment of mitral annular dimensions by three-dimensional speckle-tracking echocardiography in apical 4-chamber (A) and 2-chamber (B) long-axis views and in a short-axis view (C7) at the level of the mitral annulus in a representative case**. LV, left ventricle; RA, right atrium; LA, left atrium; RV, right ventricle; Circ, perimeter; TA, tricuspid annulus; MA, mitral annulus.

#### 3.3.2 In Lipedema

Insights from the MAGYAR-Path study, lipedema was confirmed to be characterized by dilation of end‐diastolic and end‐systolic MA diameters, areas, and perimeters and associated MA functional impairment represented by reduced MA functional area change. The short-term application of MCSs did not appear to mitigate these morphological or functional alterations [30].

### 3.4 Aortic Valve (AV)

#### 3.4.1 Normal Physiological Conditions

The AV functions as the interface between the LV outflow tract and the ascending aorta. Comprising three delicate semilunar cusps, it ensures unidirectional flow into the systemic circulation by opening during systole to facilitate ejection and closing during diastole to prevent regurgitation into the LV [45,46,47].

#### 3.4.2 In Lipedema

In middle-aged lipedema patients without cardiovascular symptoms, no valvular alterations, including significant valvular regurgitation or stenosis, could be detected on any valves [26,27,28,29,30,31].

### 3.5 Aorta

#### 3.5.1 Normal Physiological Conditions

As the body’s primary artery, the aorta transports blood from the LV to the rest of the systemic circuit. It functions as an elastic reservoir (the Windkessel effect), which is essential for maintaining steady blood flow. If the aorta loses elasticity (aortic stiffness), it can lead to higher systolic pressure, lower diastolic pressure, and increased workload for the LV. This added stress may result in LV hypertrophy, impaired relaxation, diastolic dysfunction, and compromised blood flow to the coronary arteries [45,48,49].

#### 3.5.2 In Lipedema

Mean systolic and diastolic aortic diameters were found to be increased in lipedema patients, accompanied by a higher aortic stiffness index and reduced aortic strain and distensibility compared to matched controls [31]. In contrast, another study found no significant differences in pulse wave velocity compared to age- and blood pressure-adjusted standard values, regardless of the disease stage [50]. These discrepant findings may be attributed to methodological differences, as well as variations in body habitus, body mass index, and hemodynamic factors, among others.

## 4. Pathophysiological Background

 LV rotational and LV/LA deformation abnormalities may be attributed to the enhanced fluid sequestration capacity of the highly vascularized lipedematous adipose tissue and the interlobular septa. The potential roles of an expanded population of non-cardiomyocytes—including mesenchymal cells—and subclinical epicardial adipose tissue deposition cannot be excluded. Moreover, subclinical epicardial fat deposition around the heart can also play a role in the findings. For the MA, the direct infiltration of the mitral valve’s fibrous annulus by lipedematous tissue cannot be excluded as a potential underlying mechanism. With advancing age, the prevalence of classic risk factors may increase, which may partially explain volumetric and functional LV and LA and valvular abnormalities. Increased aortic stiffness itself may have an effect on cardiac function, as has been confirmed in studies conducted in healthy subjects. Moreover, the function of the heart chambers and valves can deteriorate over time in a vicious cycle due to their reciprocal pathophysiological effects. Key research directions include investigating the cardioprotective role of elevated adiponectin levels in lipedema and the hemodynamic impact of chronic interstitial fluid sequestration on preload. Additionally, the potential contribution of epicardial adipose tissue to myocardial remodeling and the effects of estrogen receptor signaling on vascular tone and cardiac function warrant further exploration [1,2,3,4,5,6,7,8,26,27,28,29,30,31].

## 5. Clinical Implications

A central paradox in lipedema research is the apparent contradiction between a relatively favorable cardiometabolic profile and the structural/functional cardiac alterations identified by advanced imaging. Lipedema is characterized by a gynoid fat distribution, which—unlike visceral obesity—is consistently associated with preserved insulin sensitivity, lower low-density lipoprotein and triglyceride levels, and a reduced prevalence of hypertension and type 2 diabetes. This ‘protective’ phenotype, potentially linked to specific vascular endothelial growth factorgenetic variants, suggests that lipedema patients should theoretically exhibit lower cardiovascular risk. However, the findings of the MAGYAR-Path Study—such as altered LV rotational mechanics and LA enlargement—demand a careful interpretation. It remains a subject of debate whether these changes represent genuine subclinical remodeling independent of metabolic risk, or rather a functional hemodynamic adaptation. The chronic interstitial fluid sequestration and altered lymphatic drainage characteristic of lipedema may lead to a persistent shift in volume distribution, impacting cardiac preload and potentially manifesting as the observed echocardiographic anomalies. Whether these alterations are reversible following targeted lipedema treatment or represent a permanent structural shift is currently unknown, but this tension remains the primary focus of ongoing clinical investigations [1,2,3,4,5,6,7,8,26,27,28,29,30,31].

## 6. Conclusions

To date, the MAGYAR-Path Study remains the only research program to have echocardiographically investigated lipedema-associated left heart abnormalities. By addressing this gap in the literature, it serves as a foundation to motivate and guide future research in the field. Although lipedema is a known and widely examined disorder, in recent studies significant abnormalities of the dimensions and functions of the LV, LA, MA and the aorta could be demonstrated (Table 1, Ref. [26,27,28,29,30,31,50]). It has also been proven that the short-term use of MCSs can have varying degrees of effects on the heart chambers and valves. However, the currently available literature data remain limited, with substantial gaps in the literature, therefore further studies are warranted.

**Table 1.** **Summarization of the most important findings**.

Cardiovascular structure	Main finding	Sample size	Study design	Statistical significance	References
Left ventricle	In some lipedema cases, near-complete absence of LV twist (n = 1), counterclockwise LV-RBR (n = 1) and reversed LV rotations (n = 1) could be detected.	study sample: n = 25; analyzable sample: n = 22; event count: n = 1 for each abnormal LV rotational pattern	cross-sectional single-center cohort	-	[26]*
	In lipedema patients with normally directed LV rotational mechanics, reduction of apical LV rotation and consequent LV twist could be observed with preserved basal LV rotation.	study sample: n = 25; analyzable sample: n = 22; patients with normal LV rotational mechanics: n = 19	cross-sectional single-center cohort	*p* < 0.05	[26]*
	Following the 60-minute application of MCSs, lipedema patients with physiological rotational patterns exhibited a significant reduction in basal LV rotation, accompanied by a significant compensatory increase in apical LV rotation. As a result, overall LV twist remained unchanged.	study sample: n = 23; analyzable sample: n = 20; patients with normal LV rotational mechanics: n = 14	cross-sectional single-center cohort	*p* < 0.05	[27]*
	In patients with lipedema, elevated LV-CS and LV-AS may represent a compensatory mechanism for maintaining systemic pumping function. Furthermore, the short-term application of MCSs appears to have no significant global impact on LV myocardial deformation.	n = 19	cross-sectional single-center cohort	*p* < 0.05	[28]*
Left atrium	Lipedema is characterized by significantly increased LA volumes and distinct alterations in LA functional properties. Furthermore, the application of MCSs exacerbates the increase of specific LA volumetric parameters.	n = 25	cross-sectional single-center cohort	*p* < 0.05	[29]*
Mitral valve	The MA is dilated and functionally impaired in lipedema patients, the use of MCSs does not improve these alterations.	n = 24	cross-sectional single-center cohort	*p* < 0.05	[30]*
Aortic valve	Randomly selected lipedema patients showed no significant valvular regurgitation or stenosis.	n = 24	cross-sectional single-center cohort	*p* = ns	[30]*
Aorta	Patients with lipedema show dilated aortic dimensions and increased aortic stiffness.	n = 14	cross-sectional single-center cohort	*p* < 0.05	[31]
	No significant difference in pulse wave velocity compared to published standard values adjusted to age and blood pressure was found in lipedema regardless of the stage of the disease.	n = 41	cross-sectional single-center cohort	n = ns	[50]

*presents findings from the MAGYAR-Path Study. AS, area strain; CS, circumferential strain; LA, left atrial; LV, left ventricular; MCS, medical compression stocking; RBR, rigid body rotation; MAGYAR-Path, Motion Analysis of the heart and Great vessels bY three-dimensionAl speckle-tRacking echocardiography in Pathological cases; ns, non-significant.
